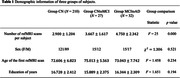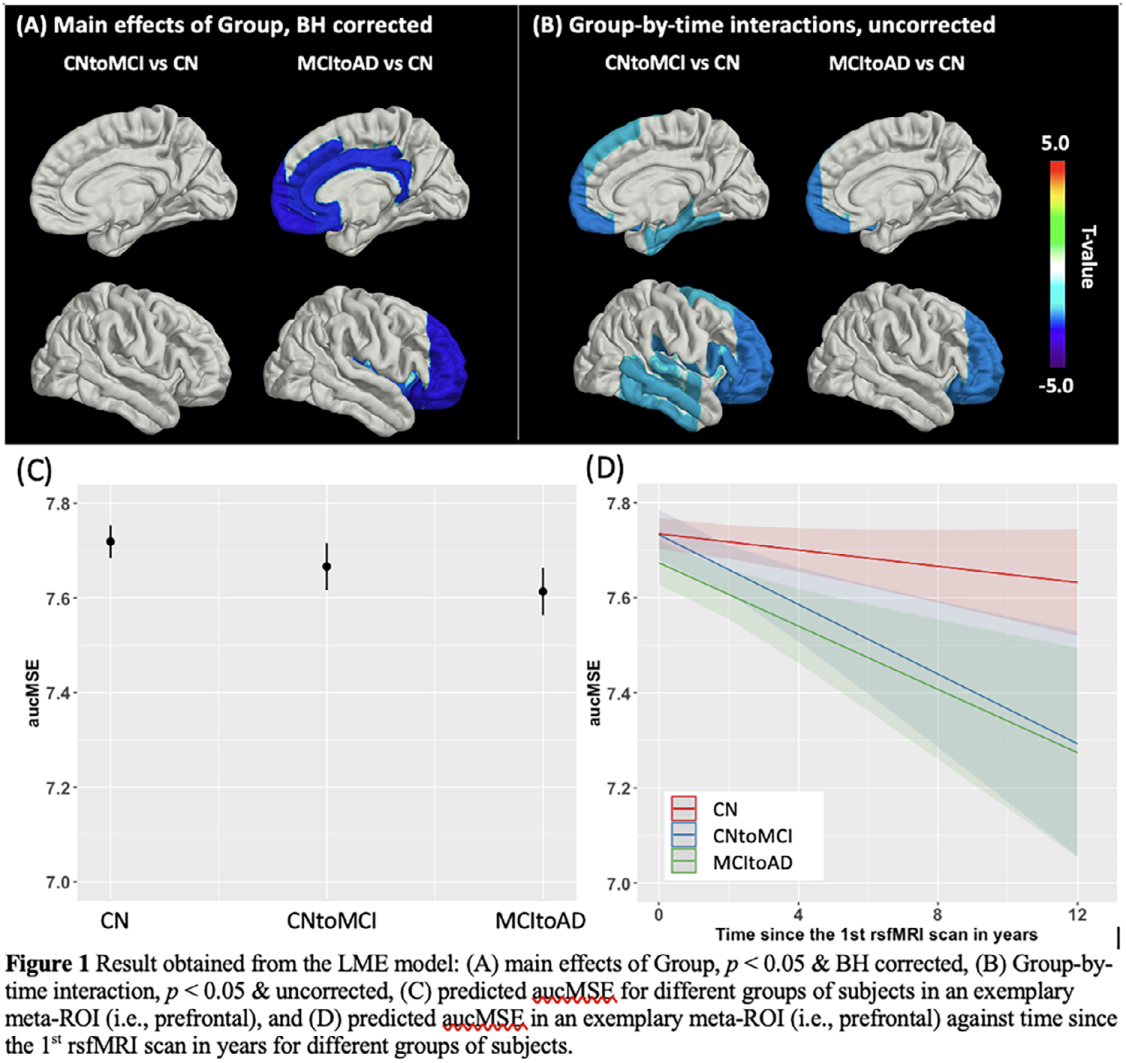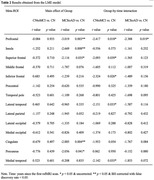# Progression of functional brain complexity in longitudinal Alzheimer's diseases

**DOI:** 10.1002/alz.086350

**Published:** 2025-01-09

**Authors:** Ru Zhang, Leon M. Aksman, Dilmini Wijesinghe, John M Ringman, Danny JJ Wang, Kay Jann

**Affiliations:** ^1^ Mark and Mary Stevens Neuroimaging and Informatics Institute, University of Southern California, Los Angeles, CA USA; ^2^ Memory and Aging Center, University of Southern California, Los Angeles, CA USA

## Abstract

**Background:**

Reduced complexity of resting‐state fMRI has been associated with mild cognitive impairment (MCI) and Alzheimer's diseases (AD) in cross‐sectional cohorts. However, the trajectory of complexity in AD progression remains unknown. We conducted complexity analyses in a longitudinal AD dataset.

**Method:**

We used demographic, clinical, T1 structural, and resting‐state fMRI (rsfMRI) data from the Alzheimer’s Disease Neuroimaging Initiative (ADNI) database. The final sample included 210 subjects in Group CN (remaining cognitively normal), 27 subjects in Group CNtoMCI (converted from CN to MCI), and 32 subjects in Group MCItoAD (converted from MCI to AD). The three groups matched in terms of sex, education, and age of their first rsfMRI scan (Table 1).

Standard image preprocessing was performed in the CONN toolbox. Multiscale entropy (MSE) for number of temporal scales *a* = 6 (0.33‐0.05Hz) was computed for each of fourteen meta regions of interest (meta‐ROIs). The area under a curve across all scales was calculated to reflect complexity for each meta‐ROI.

A linear mixed effects (LME) model was implemented to evaluate group differences in complexity and altered rates of progression of complexity across groups.

**Result:**

The LME model revealed significantly lower rsfMRI‐complexity in Group MCItoAD as compared to Group CN in the prefrontal, cingulate, and insula (*t* = ‐3.019 to ‐2.669, *p* = 0.003 to 0.008, Benjamini‐Hochberg (BH) corrected with false discovery rate < 0.05, Figure 1(A), 1(C), & Table 2). Additionally, rsfMRI‐complexity decayed significantly faster in Group CNtoMCI than in Group CN in the prefrontal, superior frontal, inferior frontal, lateral temporal, and medial temporal lobe (*t* = ‐2.417 to ‐2.093, *p* < 0.05, uncorrected, Figure 1(B), 1(D), & Table 2). By contrast, complexity decayed significantly faster in Group MCItoAD relative to Group CN only in the prefrontal (*t* = ‐2.338, *p* < 0.05, uncorrected, Figure 1(B), 1(D), & Table 2).

**Conclusion:**

The affected regions were mainly the frontal and temporal cortices which was consistent with the hypothesis of decline in executive functions and memory in AD. It demonstrates the potential of complexity analysis as an early AD biomarker.